# Poor control of interference from negative content hampers the effectiveness of humour as a source of positive emotional experiences

**DOI:** 10.1038/s41598-019-44550-3

**Published:** 2019-05-29

**Authors:** Ilona Papousek, Helmut K. Lackner, Bernhard Weber, Corinna M. Perchtold, Andreas Fink, Elisabeth M. Weiss

**Affiliations:** 1 Section of Biological Psychology, Institute of Psychology, University of Graz, Graz, Austria; 2 Division of Physiology, Otto Loewi Research Center, Medical University of Graz, Graz, Austria

**Keywords:** Cognitive control, Emotion, Cardiovascular biology, Human behaviour

## Abstract

The brain-based ability to direct attention away from interfering negative information may co-determine to which degree one may benefit from humour as a source of positive emotional experiences. This should be particularly relevant when it comes to humour that implicates a target the joke makes fun of, which inherently entails rivalry between positive and negative emotional representations. One hundred healthy individuals completed a pictorial negative affective priming task and a nonverbal humour processing task. In line with the notion that during the elaborative processing of malicious jokes, interference from negative emotional representations hampers the experience of amusement, participants took more time to judge their amusement evoked by malicious compared to benign jokes. Lesser ability to distract attention from interfering negative emotional representations was associated with slower judgements of amusement following the processing of malicious jokes, as well as with lower amusement ratings. The time it took participants to comprehend the punch-lines was not affected, neither was the immediate, short-lived pleasure after having comprehended the humour, measured by characteristic transient cardiac activation. The findings suggest that the effective use of humour as a source of positive emotional experiences requires the ability to overcome the dark side of typical humour.

## Introduction

Positive emotional experiences in one’s daily life seem to serve various beneficial purposes beyond hedonic pleasure. They may help to provide a momentary respite from ongoing stress and adversity^1–3^, and may perturb downward spirals of chronic negative moods and stress perhaps even in emotion-related psychopathology^4^. Some research reported that positive emotional experiences such as short-lived joy or amusement may exert transient analgesic-like effects^5–8^. However, even when exposed to the same amount of positive emotion elicitors in their daily lives, some people benefit more than others. This seems to be attributed to individual differences in positive emotional responsiveness, that is, the propensity to respond with elated mood or amusement to pleasant everyday events. Research indicated that positive emotional responsiveness represents a relevant disposition in the context of successful functioning in spite of adversity, and even in the recovery from affective disturbances^3,9–13^.

Various types of events, either occurring by accident or intentionally sought, can be sources of positive emotional experiences. One potential source is humour. The predominant emotional response to the perception of humour is amusement, which is defined as a facet of joy or happiness^14^. The positive emotional experience elicited by humour is linked with activation of reward related brain circuitry, which is more strongly activated the more amusement the receiver perceives^15–20^. Compared to other, less controllable elicitors of positive emotional experiences, humour has the advantage of being easily accessible, practically at any time. However, the magnitude and duration of the positive emotional experience elicited by humour varies greatly inter-individually^21,22^, which makes the clarification of moderating factors an important issue. The present study addresses the question whether affective interference processing may be one individual differences factor co-determining the effectiveness of humour as a source of positive emotional experiences. Specifically, this concerns the brain-based ability to direct attention away from interfering negative (sad) information or mental representations.

According to psychological theories of humour appreciation^23–26^, typical humour first involves the perception of a situation or event in two incompatible associative contexts. The sense of having comprehended the humour arises when this surprising incongruity is suddenly resolved by consideration of additional information available elsewhere in the joke or distant associative links, and coherence is re-established by reinterpretation of the incongruent information. The sudden comprehension of the punch-line is a pleasurable experience as such^27^ and is followed by humour elaboration. The elaboration stage involves cognitive elaboration of the reinterpretation that led to the perception of the punch-line and of its implications, and the generation of inferences about attributes of the protagonists in the humorous material or situation. These thoughts do not necessarily concern (only) the humour-eliciting features but may also involve negative or judgmental thoughts, which may diminish the final experience of amusement^26^. Taken together, two consecutive processes play a role in the positive emotional experience elicited by humour. First, the comprehension of the joke, that is, the sudden perception of the punch-line produces a short-lived pleasant feeling. The subsequent elaboration process results in the final conscious perception and evaluation of one’s amusement, and as such determines the magnitude and duration of the positive emotional experience. Targeted neuroscientific research supported the consecutive operation of these two processes^28–30^.

Importantly, negative evaluations and associated negative feelings during humour elaboration in part result from the typical structure of the incongruity-resolution type of humour alone. Most of this humour does not only include positive but also negative emotional aspects, because the joke is made at the expense of a “victim” (the target of the joke). Typically, the perspectives of two protagonists are involved in a joke, of which one is positive and one (the target’s one) is negative. Consequently, most jokes do not only spontaneously evoke amusement but at the same time also negative feelings such as vicarious sadness, embarrassment or pity^31^. To which degree the perception of humour finally results in a positive emotional experience is presumably influenced by the extent to which such negative emotional aspects figure in the individual’s elaboration of the humour.

In the case of malicious humour, the inherent rivalry between positive and negative emotional representations during humour elaboration should make it more difficult to perceive one’s level of amusement. Moreover, this difficulty should be more pronounced the less the individual is able to ignore or inhibit the negative components, which interfere with the task of perceiving and judging one’s own amusement. There is some preliminary evidence from previous research that used a similar humour task as in the present study, showing that individuals with greater difficulties to control negative information took more time to rate their amusement elicited by the humour. In one of these studies, a neurophysiological indicator for poorer ability to down-modulate negative social-emotional perceptual input was associated with slower responses to the amusement ratings^32^. Another study showed that individuals with poorer self-reported regulation of negative emotions in everyday life, which captured basic automatic processes including inhibitory processes as well as volitional emotion regulation efforts, took more time to deliver the amusement ratings^33^. This was particularly true for cartoons implying a social situation, in which most of the punch-lines were at the expense of one of the protagonists in the cartoon (i.e., of which most implied a classic victim). Finally, it was demonstrated that response latencies were slower in individuals with higher social anxiety, which is associated with greater sensitivity to negative social-emotional information^34^. Again, this was shown for the cartoons illustrating a social story in particular. In some of these studies, participants also reported lower amusement levels. Studies using other humour tasks showed, for instance, that the experience of amusement was reduced in individuals who identified themselves with the target of the joke to a greater degree^31,35,36^. Furthermore, the activation of reward areas by humour was moderated by neuroticism, which is related to increased sensitivity to negative information^37^.

However, those previous studies provided only preliminary and indirect evidence for the relevance of individual differences in the ability to ignore or inhibit interfering negative emotional components to the positive emotional experience derived from humour. In the present study, we used a negative affective priming (NAP) paradigm, which more directly assesses the brain-based ability to ignore distracting negative (or positive) emotional information, i.e., affective interference control^38,39^. Following Goeleven *et al*.^38^, facial expressions (rather than emotional words) were used as stimuli. Faces are important social communication cues and as such convey social-emotional information. Individual differences in the ability to control interference from facial emotional expressions may, therefore, be particularly relevant to the biased interpretation of ambiguous social situations^40^, which are also an integral component in typical humour. Importantly, the NAP task predominantly taps biases at later, elaborative stages of information processing, such as difficulties to disengage from or inhibit emotional information, rather than biases in the early selection of or orientation toward negative or positive information^40^.Therefore, it should be especially relevant in the present context. In order to detect the punch-line, it is necessary to also process the negative information (which is vital for detecting and resolving the incongruity). Only then, during humour elaboration and judgement of one’s amusement, the negative components become distractors that interfere with the perception of amusement and, hence, a sustained positive emotional experience.

In the humour processing task, participants were presented with a series of nonverbal, visual jokes. After viewing each picture, the participants indicated whether they did or did not comprehend the punch-line and rated their amusement. Responses to typical, “malicious” humour, implicating a target the joke makes fun of, were contrasted with more benign humour. The response latency to the comprehension question is primarily determined by the cognitive process of detecting the humour (i.e., the easiness of detecting the punch-line^33,34^). The immediate pleasure of having comprehended the humour is reflected in characteristic transient cardiac activation. This Contrasted Transient Response (CTR) is determined by studying the dynamics of the heart rate during a time window immediately before the participants indicated having recognised the punch-line, contrasting it to the activation during the processing of matched non-humorous material^27,32^. The difficulty to properly perceive and judge one’s level of amusement manifests in a further objective behavioural measure, i.e., slower response latencies to the amusement rating, and may also manifest in lower amusement ratings^32,34^.

We expected (1) that, due to interference from negative emotional representations, participants would have more difficulties perceiving their amusement following the processing of humour implying a classic “victim” compared to more benign jokes. This should result in longer response latencies while judging the evoked amusement when the punch-line is at the expense of a target. We further expected (2) that in the case of malicious jokes, lesser ability to control interference from negative emotional information as assessed by the NAP task would slow down the judgement of amusement. (3) More negative appraisal of the scenarios depicted in the cartoons in participants with dysfunctional control of interfering negative emotions should also result in less positive emotional experiences, that is, lower amusement levels. (4) As elaboration has not yet taken place at the time of the immediate pleasure of having comprehended the humour, we did not expect dysfunctional control of negative emotions in the NAP task to affect the cardiac CTR. (5) To allow for more specific conclusions, control of interfering positive emotional representations was also tested for comparison. We did not have a particular hypothesis with regard to the effects of individual differences in the processing of interference from positive emotional information.

## Results

### Slowed down judgement of amusement

For testing the main research questions, a general linear model was used with the response latency to the amusement rating in the humour processing task as the dependent variable, type of cartoon (malicious vs. benign) as within-subjects factor and the NAP scores for sad (NAP-sad) and happy (NAP-happy) targets as two continuous between-subjects factors. The relevant effects in this analysis are the main effect of type of cartoon (1^st^ hypothesis) and the interaction of type of cartoon by NAP-sad. The interaction effect is the crucial effect for evaluating the primary research question whether in the case of exposure to typical humour implicating a target the joke makes fun of, poorer control of interfering negative emotional information may slow down the judgement of amusement (2^nd^ hypothesis). The analysis revealed a main effect of type of cartoon (*F*(1,97) = 12.8, *p* = 0.001, ɳ² = 0.12), indicating that participants took more time to judge their amusement when the humour implied a classic “victim” (*M* = 1157 ms, *SD* = 285) compared to more benign jokes (*M* = 1092 ms, *SD* = 270). Further, there was a significant interaction effect of type of cartoon by NAP-sad (*F*(1,97) = 5.4, *p* = 0.022, ɳ² = 0.05). The other effects were non-significant (type of cartoon by NAP-happy: *F*(1,97) = 0.4, *p* = 0.557; main effect NAP-sad: *F*(1,97) = 0.7, *p* = 0.397; main effect NAP-happy: *F*(1,97) = 0.2, *p* = 0.703). To follow up the interaction effect for the purpose of its interpretation, standard multiple regression was used, with NAP-sad as predictor and response latency to the amusement ratings of malicious cartoons as the dependent variable. To retain the contrast with responses to more benign humour, and at the same time controlling for general response speed^32–34,41^, response latency to benign cartoons was additionally entered in the equation. The relevant semi-partial correlation between NAP-sad and response latency to the amusement ratings of malicious cartoons was *sr* = −0.13 (*p* = 0.017, zero-order correlation *r* = −0.14; *F*(2,97) = 131.6, *p* < 0.001). The result indicates that poorer control of interference from negative emotional information slows down the judgement of amusement when individuals are confronted with typical humour that is at the expense of a target.

### Perceived levels of amusement

For the analysis of the perceived level of amusement (3^rd^ hypothesis), the same statistical approach was used as for the analysis of the response latency above. The main effect of type of cartoon was non-significant (*F*(1,97) = 0.2, *p* = 0.682). The interaction of type of cartoon by NAP-sad was again significant (*F*(1,97) = 4.7, *p* = 0.032, ɳ² = 0.05; type of cartoon by NAP-happy: *F*(1,97) = 0.4, *p* = 0.533; main effect NAP-sad: *F*(1,97) = 3.8, *p* = 0.055; main effect NAP-happy: *F*(1,97) = 0.2, *p* = 0.631). In the follow-up regression analysis, the relevant semipartial correlation between NAP-sad and amusement levels was *r* = 0.12 (*p* = 0.012, zero-order correlation *r* = 0.24; *F*(2,97) = 128.1, *p* < 0.001), indicating that, when confronted with typical, more malicious humour, individuals with poorer control of interference from negative emotional information perceived less amusement. Amusement ratings and response latencies to the amusement ratings were correlated with *r* = −0.23 (*p* = 0.020).

### Immediate pleasure of having comprehended the humour

The analysis of the cardiac CTR, indicating the immediate pleasure of having comprehended the humour (4^th^ hypothesis), revealed an on average greater response to malicious (*M* = 0.59, *SD* = 2.0) compared to benign humour (*M* = 0.11, *SD* = 1.9; main effect type of cartoon: *F*(1,97) = 4.9, *p* = 0.029, ɳ² = 0.05). The interaction effect of type of cartoon by NAP-sad was non-significant in this analysis (*F*(1,97) = 0.3, *p* = 0.562). However, there was a significant main effect of NAP-happy (*F*(1,97) = 6.7, *p* = 0.011, ɳ² = 0.07; main effect NAP-sad: *F*(1,97) = 0.05, *p* = 0.828; type of cartoon by NAP-happy: (*F*(1,97) = 1.4, *p* = 0.236). Immediate cardiac responses were smaller in individuals with more effective control of interference from positive emotional information (*r* = −0.25, *p* = 0.011). Correlations between the CTR and the other indicators of the humour processing task were all non-significant (response latency to comprehension question: *r* = 0.02, *p* = 0.818; response latency to amusement rating: *r* = −0.12, *p* = 0.218; amusement level: *r* = 0.13, *p* = 0.182).

### Supplementary analyses

Higher levels of positive mood were associated with experiences of greater amusement elicited by both types of humour (*r* = 0.29, *p* = 0.004; *r* = 0.29, *p* = 0.004) as well as faster judgements of amusement (*r* = −0.20 *p* = 0.05; *r* = −0.24, *p* = 0.015). Correlations with the CTR were non-significant (*r* = 0.17, *p* = 0.094; *r = *0.15, *p* = 0.147). Moreover, higher levels of positive mood were related to more effective control of interference from negative emotional information (NAP-sad; *r* = 0.25, *p* = 0.011; NAP-happy: *r* = −0.06, *p* = 0.550). Correlations with the negative activation subscale of the PANAS were all non-significant. To evaluate the role of positive mood in the relationships of primary interest, the follow-up regression analyses were repeated with additionally entering positive mood in the equation. While the semi-partial correlation between NAP-sad and response latencies to the amusement ratings of malicious cartoons remained significant (*sr* = −0.11, *p* = 0.038), the correlation between positive mood and response latencies was abolished (*sr* = −0.05, *p* = 0.361; *F*(3,96) = 87.9, *p* < 0.001). The same picture emerged for the amusement levels (NAP-sad: *sr* = −0.11, *p* = 0.017; positive mood: *sr* = 0.011, *p* = 0.816; *F*(3,96) = 120.2, *p* < 0.001). Thus, it can be ruled out that the central correlations were solely due to the links of both variables with positive mood. Rather, it seems that the association between enhanced perception of amusement in challenging conditions and positive mood is explained by the variance in control of interference from negative emotional information.

Response latencies to the comprehension question were analysed analogous to the other parameters of humour processing, primarily to evaluate whether malicious and benign cartoons might have differed in complexity (which would manifest in differences in the required time to comprehend the punchline). No significant effects were observed. Most importantly, response times did not differ between the two types of cartoons (*F*(1,97) = 1.0, *p* = 0.311). Thus, there is no indication that malicious and benign cartoons might have differed in the complexity of their punchlines. All other effects were also non-significant (all *p* > 0.60).

Average priming effects in the NAP task were examined using a 2 (condition: experimental vs. control) × 2 (valence: sad vs. happy targets) analysis of variance. Response times were on average slower in the experimental (*M* = 814 ms, *SD* = 135) than in the control conditions (*M* = 808 ms, *SD* = 133), indicating a negative priming effect overall (main effect of condition, *F*(1,99) = 7.5, *p* = 0.007, ɳ² = 0.07). The significant interaction effect (*F*(1,99) = 12.2, *p* = 0.001, ɳ² = 0.11) along with paired samples t-tests revealed that response times in experimental trials were only slower compared to control trials with regards to happy targets (*M* = 813, *SD* = 137; *M* = 798, *SD* = 139; *t*(99) = 4.3, *p* < 0.001). Differences between response times on experimental and control trials were non-significant for sad faces (*M* = 815, *SD* = 137; *M* = 817, *SD* = 133; *t*(99) = 0.6, *p* = 0.569), pointing to an on average greater difficulty to control negative compared to positive distracting information. The correlation between the NAP effects in probe trials with happy / sad target faces was *r* = −0.01 (*p* = 0.919). Men and Women did not differ in any of the variables used in this study (independent t-tests; all *p* > 0.18).

## Discussion

The present study was concerned with the question whether an individual’s ability to direct attention away from interfering negative information or mental representations may in part co-determine to which degree he or she may benefit from humour as a source of positive emotional experiences. It was argued that the ability to control interference from negative emotional information should be particularly relevant in typical jokes that implicate a target the joke makes fun of. This type of humour may not only spontaneously evoke amusement but may also implicate negative evaluations and associated negative feelings, particularly during the elaboration process, which follows the immediate, short-lived pleasurable experience of having comprehended the joke^26,31^. The extent to which the negative emotional aspects figure in the individual’s elaboration of the humour may then determine the magnitude and duration of the positive emotional experience the individual derives from the humour. The present study focused on the elaborative stages of humour and affective interference processing, because they are more relevant to the putative implications of positive emotional experiences, which require that the positive experience persists for some time^42,43^.

If it is true that interference from negative emotional representations hampers the conscious experience of amusement following confrontation with jokes made at the expense of a target, individuals should have more difficulties perceiving their amusement when exposed to this type of humour compared to benign humour. This basic notion was confirmed by the finding that participants took more time to judge their amusement evoked by malicious compared to benign jokes. More indirectly, previous research had already shown slower response latencies to ratings of amusement when cartoons implicated social situations, which inherently entail more rivalry between positive and negative emotional representations than non-social jokes^33^. Important to note, the times it took the participants to comprehend the punch-lines did not differ between jokes implying a classic “victim” and more benign jokes. From this it follows that the differences in the response latencies to the ratings of amusement cannot be explained by higher complexity of the malicious cartoons. The percentage of detected punch-lines was also not lower for malicious compared to benign cartoons.

Further, the present results confirmed the relevance of individual differences in the brain-based ability to control interference from negative emotional information in this context. The findings indicated that when the joke was made at the expense of a target, lesser ability to distract attention from interfering negative emotional representations slowed down the participant’s judgement of his or her amusement. Moreover, amusement ratings were lower in this case, that is, emotional experiences were less positive. This is a novel finding, for previous research had provided only indirect evidence for this idea^31,33–37^. Taken as a whole, findings were stronger for response latencies to the ratings than for levels of amusement in this research. This is probably due to the fact that as the only self-reported variable derived from the humour processing task, the rating of amusement is weaker and more prone to demand characteristics than the more objective response time^32^. It can be excluded that the relationship between affective interference control with response latencies and amusement ratings to malicious humour may be explained by individual differences in general response speed or general response tendencies, because this was controlled for by the concomitant analysis of the scores for benign cartoons^32–34,41^.

We had proposed that affective interference control should only play a role in the elaborative stage of humour processing, in which the reinterpretation of information in the joke and its implications are elaborated and also negative or judgemental thoughts may arise. In line with this idea, the data did not show a moderating effect of the ability to distract attention from interfering negative information on the immediate pleasure of having comprehended the humour (indexed by the cardiac CTR). Affective interference control did also not affect how long it took the participants to comprehend the punch-lines. These findings can be integrated in previous research targeting individuals prone to unfavourable attention biases such as depressed and socially anxious individuals. Compared to healthy persons, individuals with depression or social anxiety do not seem to derive equivalent and equally sustained levels of positive affect from humour, while the detection of punch-lines and the cognitive comprehension of humour as well as the generation of immediate positive reactions to humour are unaffected^34,44,45^.

While individual differences in the processing of interference from negative emotional information did not affect the cardiac immediate pleasure response after having “gotten” the joke, these short-lived immediate pleasure responses were on average higher in the case of malicious compared to more benign jokes. This may be explained by additional incongruity that is introduced by the incompatible mental states of the protagonists in disparaging jokes. The greater the incongruity, the greater may be the immediate pleasure when it is suddenly resolved^46^. After conscious elaboration of the joke as a whole, during which also the negative aspects of the humour slip in, the total emotional experience derived from the humour is less positive in the case of more malicious humour, especially in individuals with dysfunctional control of interference from negative representations. As a result, participants with poorer interference control gave lower ratings of their amusement when jokes were at the expense of a “victim”.

An average priming effect in the NAP task was only found for happy but not for sad distractors. This is in accordance with a number of previous studies, which did not find a significant average NAP effect with negative emotional material in healthy samples either^47–51^. It seems that, on average, interference from negative information is more difficult to ignore than from positive information^50^. However, it is beyond question that individual differences in the inhibition of interference nevertheless show meaningful relationships to other variables, which can be different for negative and positive distractors^38,52^. It is also not an unusual finding that NAP indicators of interference control for negative and positive representations are uncorrelated^40^.

The present study has several limitations. While it can be ruled out that malicious cartoons were more complex or more difficult to understand, it cannot be entirely excluded that the two types of cartoons might have differed in some other cognitive or perceptual feature that might have influenced the results to some extent. All participants were high educated university students and, therefore, the results cannot necessarily be generalised to non-student populations. Further, effect sizes were small. It seems likely that larger effects may have been observed when more spiteful jokes were used (the malicious cartoons used in the present study were still relatively unoffending). However, the small effect sizes might also be explained by the somewhat less than perfect reliability of NAP scores^53^. For these reasons, replication of the findings with another suitable test for the assessment of the ability to ignore distracting information and with other, perhaps more extreme series of jokes would be desirable. Pending more powerful replication, the small-sized results are more of academic than of practical value. Future studies may also target populations with expected compromised affective interference control such as depressed^38,40,47,52,54^ or aggressive^55^ individuals.

In conclusion, it seems necessary to be able to overcome the dark side of humour for being able to effectively use humour as a source of positive emotional experiences. Functional control of interference from negative emotional representations seems to facilitate the perception of amusement, particularly when it comes to typical humour, which almost always is at the expense of a target the joke makes fun of. While it is premature to extrapolate practical consequences from this research, one recommendation might be to use exclusively benign humorous material as a source of positive emotional experiences, particularly for people with conditions that are associated with dysfunctional affective interference control such as individuals with depression or elevated depressed mood^38,40,47,52,54^.

## Methods

### Participants

The final sample comprised *n* = 100 participants (45 men, 55 women). All participants were university students enrolled in various fields. The study was advertised as a “study on factors influencing the appreciation of jokes and their physiological effects” in lectures and via e-mail sent out by the university administration. Interested participants entered their phone numbers in a list or in an e-mail to the experimenter and were called to fix an appointment. Healthy participants were tested, excluding potential confounding consequences of mental illness or medication. Individuals with major psychiatric disorders/history of major psychiatric disorders according to the SCID-I (Structured Clinical Interview for DSM-IV Axis I Disorders^56^, and individuals who reported having a neurological disease or using psychoactive medication were not included in the study. To get reliable cardiac measures, individuals who reported having a cardiac disease or using cardioactive medication were not included, as well as seriously underweight persons (BMI < 17.5 kg/m²). Individuals who had participated in a study using the humour processing task before were also excluded. The age of the participants was between 18 and 42 years (*M* = 22.0, *SD* = 3.7). The study was performed in accordance with the Declaration of Helsinki and was approved by the ethics committee of the University of Graz, Austria. Participants gave their written informed consent to participate in the study. They received course credit or € 10,- for their participation.

### Negative affective priming task

The NAP task is based on the concept of negative priming, which is a cognitive process whereby the response to a previously ignored stimulus is delayed^57,58^. In the NAP task, the negative priming effect is observed when ignoring an emotional stimulus results in a delayed response to a stimulus of the same valence that is presented as the target in a subsequent trial. These slowed down responses indicate inhibition of attention to interfering emotional representations. The reverse pattern (relatively faster responses or positive priming) indicates deficient inhibition or facilitation. NAP tasks have been widely used in the past with either emotional words or pictures of facial expressions as stimuli^38,40,47–49,52,59–64^.

Based on the pictorial negative affective priming (NAP) paradigm developed by Goeleven *et al*.^38^, a computerized task was created using 30 sad, 30 happy, and 20 neutral pictures selected from the Karolinska Directed Emotional Faces (KDEF) database^65,66^. In each trial of the NAP task, participants were presented with two pictures of facial expressions and were instructed to indicate whether the target face (marked by a black frame) displayed a negative or a positive emotion, while ignoring the distractor face (marked by a grey frame). Trials began with the presentation of a centred fixation cross (1000 ms), followed by the presentation of two vertically arranged pictures displaying sad or happy expressions (prime trial). Prime trials were followed by test trials, in which an emotional face (sad or happy) was presented together with a neutral one. In the “experimental” test trials, the emotional expression of the target picture was the same as the emotional expression of the previously ignored picture in the prime trial (one sad and one happy expression was displayed in those prime trials). In the “control” test trials, the emotional expression of the target picture differed from the emotional expression of the previously ignored picture in the prime trial (either two sad or two happy expressions were displayed in prime trials preceding control test trials). See Goeleven *et al*.^38^ for a schematic representation of the composition of trials. The task included a total of 256 prime and test trials, respectively. Participants made responses to all (i.e., prime and test) trials, which did not appear different to them, but only response times to the test trials were relevant and therefore analysed. There was a 1000 ms blank screen interval between trials. Participants completed an equal number of trials with the target face in the top and bottom position, and the stimuli were presented in a fixed random order.

Response times only entered the analysis if responses were correct in both trials of a prime–test trial pair. This was the case for *M* = 92.78% (*SD* = 5.01) of all prime–test trial pairs. Following standard procedures, extreme response times (below 300 ms and above 2000 ms) were considered outliers and eliminated from the analysis. For the analysis of individual differences in interference processing, NAP scores were computed by subtracting the mean response time on control test trials from the mean response time on experimental test trials. This was performed separately for test trials with sad and happy targets^40,47–49,52,61,62,64^. A positive value of the NAP score indicates negative priming (inhibition). A negative value indicates deficient inhibition or facilitation (positive priming).

We opted for the NAP task rather than the Stroop task, which is commonly being used for the assessment of similar mechanisms, because the NAP task captures a later, more lasting inhibition-dominated process, whereas performance in the Stroop task is dominated by the process related to immediate stimulus selection^38,50,67^.

### Humour processing task

The computer-based task comprised 60 humorous cartoons, which were mixed with 20 non-humorous pictures that did not contain a punch-line. All cartoons were non-verbal line drawings and contained an incongruity that could be resolved meaningfully (i.e., they followed a typical joke pattern). Cartoons were classified by the first two authors as typical, “malicious” humour, if the punch-lines implied some negative experience of the target (*n* = 28). The remaining 32 benign cartoons were used as the reference condition in the analyses. The non-humorous pictures were needed for identification of the cardiac response (see below). None of the cartoons were based on ethnic or national stereotypes or sexual content. Pictures were presented in random order. Participants were instructed to indicate via mouse click whether they had or had not recognised the punch line and to click the respective button as soon as they had recognised the punch line or were sure that they did not find it. On average, participants detected *M* = 24.3 (*SD* = 3.3) or 86.9 percent of the punch-lines in malicious cartoons and *M* = 27.0 (*SD* = 3.8) or 84.4 percent of the punch-lines in benevolent cartoons. The pictures were presented until the button was clicked (max. 6 s), followed by max. 4 s in which the participants rated their amusement on a scale ranging from 1 to 6, and another 10 s in which a fixation cross was presented, before the next picture appeared on the screen. Response latencies were averaged across all detected punch-lines in malicious and benevolent cartoons, respectively. None of the participants came near the allotted time limits (maximum response latencies to the comprehension question and the amusement rating were 4.1 s and 2.0 s, respectively; *M* = 3.1, *SD* = 0.4; *M* = 1.1, *SD* = 0.3). The paradigm was used in several previous studies, and the cartoons were selected from the pool used in those studies^27,32–34,41,46,68^.

For obtaining the cardiac Contrasted Transient Response (CTR^27^), the electrocardiogram was recorded using portable devices (eMotion Faros 180°^TM^ (1-Channel ECG, sampling rate 1000 Hz). The disposable electrodes were placed at the thoracic region (corresponding to Einthoven lead II). Heart rate changes relative to the 0.5 s frame preceding the picture onset were separately averaged across trials with malicious/benign cartoons and trials with non-humorous pictures. The CTR was calculated as the difference between the heart rate change in the 0.5 s frame immediately preceding the participant’s response indicating having detected the punch line in malicious/benign cartoons minus the data from the same 0.5 s frame in trials with non-humorous pictures. The parameter is based on the fact that transient activation of the behavioural approach system (produced, e.g., by signals of impending reward or actual pleasure) is accompanied by transient heart rate acceleration, which comes out especially clearly when contrasting it to the response to neutral stimulation^69,70^. Higher positive values indicate more immediate pleasure related to the satisfaction derived from the sudden insight when detecting the humour. Please see Lackner *et al*.^27^ for the details how to calculate the CTR and its validity and see also Papousek *et al*.^32^, and Rominger *et al*.^71^ for applications of the CTR extending beyond that.

### Additional measures

To enable the evaluation of potential influences of current mood on the humour processing task, the negative and positive activation subscales of the Positive and Negative Affect Schedule (PANAS; German version^72^) were used. Participants rated their current affective states before they completed the humour processing task and the NAP task.

## Data Availability

The datasets generated during and/or analysed during the current study are available from the corresponding author on reasonable request.
